# Design and Characterization of Hybrid Multilayer Structures: Layer-by-Layer Growth of Polymer and Graphene Oxide Assemblies and Their Utility in Fuel Cell Applications

**DOI:** 10.1021/acsaem.5c03672

**Published:** 2026-01-13

**Authors:** Neelanjana Mukherjee, Nancy S. Muyanja, Yunzhu Zhang, Phuong Quynh Ngo, Anusorn Kongkanand, G. J. Blanchard

**Affiliations:** † Michigan State University, Department of Chemistry, 578 S. Shaw Lane, East Lansing, Michigan 48824, United States; ‡ Michigan Center for Materials Characterization, University of Michigan, College of Engineering, Ann Arbor, Michigan 48109, United States; § Fuel Cell Businesses, General Motors, Pontiac, Michigan 48340, United States

**Keywords:** fuel cell, poly(ethylene imine), graphene oxide, multilayer growth, surface modification

## Abstract

We report the layer-by-layer growth of poly­(ethylenimine) (PEI) that has been modified with sulfamate functionalities (S-PEI) using Zr^4+^-complexation interlayer linking chemistry. We have also deposited adlayers of graphene oxide (GO) that have been modified to possess sulfate functionalities (S-GO), onto the S-PEI layers. The multilayer assemblies are formed with sulfamate/sulfate and sulfate/sulfate (S-PEI + S-GO) interlayer linking chemistry. In all cases the adlayer thickness is consistent with predictions based on van der Waals volume and/or molecular mechanics calculations. X-ray photoelectron spectroscopy (XPS) is used to characterize the Zr/S stoichiometry in the multilayer assembly. The utility of these hybrid multilayer structures is demonstrated in a Proton Exchange Membrane (PEM) fuel cell, where they are shown to reduce H_2_ gas crossover by 15% with only a 12 nm thick layer.

## Graphical abstract

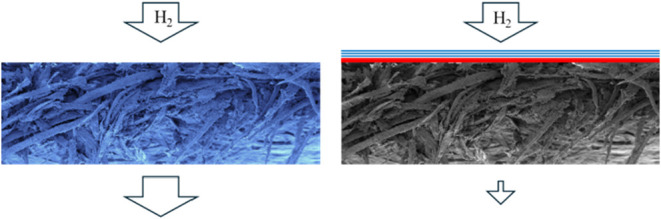

## Introduction

Poly­(ethylenimine) (PEI) is a polymeric amine that can exist either as a linear structure (LPEI) or a branched structure (BPEI). BPEI is produced commercially by the acid-catalyzed polymerization of aziridine, involving propagation through cyclic immonium cations, and the product contains primary, secondary, and tertiary amino groups in a ratio of ∼1:2:1.^1^ The high density of amino and imino groups in PEIs makes them highly water-soluble and enables their usage in a range of application areas, including as chelating agents for metal ions,^1–3^ in wastewater treatment,^4^ or as a flocculation aid in the pulp and paper industry.^4^ PEIs have also gained substantial application in novel drug delivery systems, driven by the ability to modify these polymers and in biochemistry to study their physiological action in depth.^5,6^

PEI can take on significant positive charge, as every third atom is an ionizable nitrogen. Accordingly, PEI exhibits surfactant-like properties, that can lead to disruption of lipid bilayers by pore formation and membrane erosion or thinning.^7^ Molecular weight, polydispersity, polymer structure, and extent of branching all play roles in determining the cationic charge density of the polymer. In addition, the resistance of PEI to biodegradation precludes rapid elimination and therefore contributes to cellular toxicity.^8^ Significant effort has thus been made to design PEI systems that impart biodegradability and prevent nonspecific interactions with unintended targets.^9–11^

Graphene oxide (GO) is likewise a material with a wide range of applications, albeit focused in different areas, such as electronic devices,^12,13^ supercapacitors,^14,15^ desalination membranes,^16,17^ composite materials,^17–21^ electrocatalysts,^22^ catalyst supports,^23^ gas sensors^24^ and a variety of medical therapies.^25^ Brodie first described the synthesis of GO, but Hummers is widely credited with bringing research on GO to the fore during the past two decades.^26,27^ GO is characterized by sheets that are a single atomic layer thick, with a variety of oxygen-containing functionalities, such as epoxide, hydroxyl, and carboxyl groups, resulting in useful interfacial properties. Despite numerous studies of GO surface chemistry, the distribution of functionalities on individual GO sheets remains to be fully understood, and this situation is exacerbated by its relatively unstable nature.^28^

The design and synthesis of multilayer structures has enjoyed wide interest because of the utility of such structures. We have reported previously on the formation of GO multilayers using Zr-bisphosphate and Zr-disulfate interlayer linking chemistry.^29,30^ In that work, the dimensions of the GO moieties produced multilayers where the lateral uniformity could be a limiting factor for coverage on nonuniform supports. Forming hybrid multilayer structures with GO and PEI overcomes this limitation and opens the door for potential use in nanoscale technologies ranging from catalysis through electronic devices as well as in more macroscopic applications, such as fuel cells, which we consider in this work. Among the several methods available for the formation of multilayer assemblies, layer-by-layer (LbL) deposition with robust ionic interlayer linking chemistry has proven to be a versatile choice because of the combination of functional groups and metal ions that can be used, and the inherent control over the chemical identity of each deposited layer. Layer-by-layer growth produces materials that can have significant practical advantages over polymer materials deposited by traditional bulk spin coating methods, primarily for reasons of better control over the thickness of ultrathin (∼nm) polymer layers. LbL deposition also avoids the formation of larger-scale defects, including bubbles or pinholes, and the overall composition of the assembly can be controlled much more precisely. In this paper, we consider the formation of hybrid multilayers of PEI and GO on silicon and silica surfaces using assemblies formed with sulfamate/sulfate and sulfate/sulfate (S-PEI + S-GO) interlayer linking chemistry. We also apply this same hybrid LbL chemistry to nonuniform interfaces used as a fuel cell catalyst support (*vide infra*).

## Experimental Section

### Reagents and Materials

Graphite, sodium nitrate (NaNO_3_, ≥99.0%), sulfuric acid (H_2_SO_4_, 95.0–98.0%), potassium permanganate (KMnO_4_, ≥99.0%), zirconyl chloride octahydrate (ZrOCl_2_·8H_2_O, 98%), anhydrous acetonitrile (CH_3_CN anhydrous, 99.8%), chlorosulfonic acid (ClSO_3_H, 99%), chloroform (CHCl_3_, ≥99.8%), polyethylenimine (branched), methanol (CH_3_OH anhydrous, 99.8%) and ethanol (>99.5%) were purchased from Sigma-Aldrich. Hydrogen peroxide (30%, aqueous solution) was purchased from Fisher Scientific. All reagents were used as received and were not purified further. Silicon wafers were purchased from University Wafer Inc. Silica slides were purchased from UQG Ltd. Milli-Q water (18 MΩ·cm) used in all experiments was generated using a Thermo Scientific Genpure system. Glassware that was not used in anhydrous syntheses was rinsed with Milli-Q water prior to use. Fuel cell membranes used are 12 μm thick perfluorosulfonic acid membrane (PFSA, EW = 800–820) with expanded poly­(tetrafluoroethylene) reinforcement layer. High-surface-area carbon black supported Pt nanoparticles (Pt/HSC, 40 wt %) were used as both cathode and anode electrocatalysts. The catalysts were mixed with PFSA ionomers and coated on 160 μm thick water-proof carbon paper-based gas diffusion layer to prepare gas-diffusion electrodes (GDE).

### GO and S-GO Synthesis

The GO used here was synthesized using a modification of the Hummers method that we have used previously.^26,29,30^ Graphite (0.5 g) was mixed with 23 mL of H_2_SO_4_ (conc. reagent) and stirred at 0 °C. NaNO_3_ (0.5 g) was added next, and then KMnO_4_ (3 g). The mixture was heated to 35 °C for 2 h while stirring, followed by cooling in an ice bath and 55 mL water was added at a rate that maintained the temperature of the reaction mixture below 10 °C. Five mL of H_2_O_2_ was added slowly until gas evolution was no longer observed. The resulting mixture was then vacuum filtered and the recovered solid was dispersed in 25 mL CH_3_CN (anh.). S-GO was synthesized from GO (in CH_3_CN) by adding ClSO_3_H (333 μL). The reaction mixture was stirred for 10 min. The resulting S-GO stock solution was stored in a sealed vessel until use.

### S-PEI Synthesis

PEI was sulfamated by the addition of 2.5 mL of ClSO_3_H in anhydrous CH_3_OH. The precipitate formed was filtered and dissolved in H_2_O. The S-PEI solution was stored in a sealed vessel until use. The S-PEI product was characterized using FTIR and the spectra match literature reports (Figure S1).

The sulfamation of PEI is shown schematically in Figure 1. After the functionalization of the polymer with sulfamates, the precipitate was washed with MeOH and dissolved in H_2_O before use. Sulfonation of the polymer was spontaneous and functionally instantaneous, and the S-PEI product dissolved readily in H_2_O.

**Figure 1:**
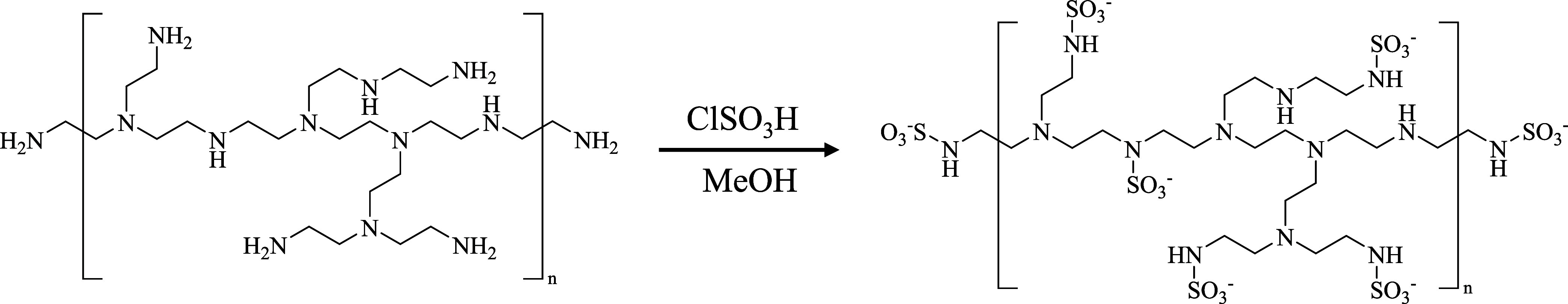
Modification of PEI to add sulfamate functionality.

### Surface Preparation

Silica and silicon substrates were cleaned by 10 min immersion in piranha solution (3:1 H_2_SO_4_/H_2_O_2_. *Caution: strong oxidizer!*), and then rinsed with Milli-Q water and dried using a stream of N_2_ (g) before layer deposition.

### Layer Deposition

Layer deposition of S-PEI is schematized in Figure 2. The silica and oxidized silicon substrates were directly sulfated using ClSO_3_H in chloroform in a fume hood. After a 10 min reaction time the substrates were rinsed by immersion in Milli-Q water and then used. Substrates were reacted to deposit Zr^4+^ by immersing them in a 60% aqueous ethanol solution of ZrOCl_2_ (5 mM) for 5 min. The resulting interfaces were immersed in the solution of S-PEI for a reaction time of 10 min, then were washed with water, dried with N_2_ and another layer of the polymer could be deposited in the same manner if multilayers of PEI are required. For the S-GO layer formation, the S-PEI adlayer was reacted with Zr^4+^ by immersion in in a 60% aqueous ethanol solution of ZrOCl_2_ (5 mM) for 2 min, then was immersed in a ∼0.2 M S-GO solution for 4 min. The resulting reacted surfaces were rinsed with CH_3_CN (anh.)­and then with water and dried with N_2_(g) prior to characterization. Deposition of multiple sequential layers of S-GO followed the same cycle.

**Figure 2:**
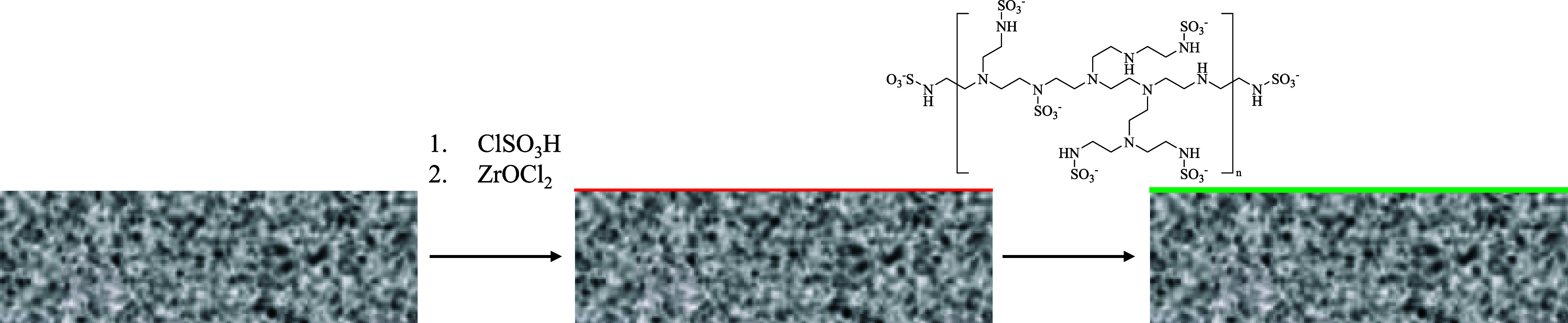
Deposition of the sulfamated polymer onto a sulfate-functionalized support, using Zr^4+^ interlayer linking chemistry.

### Fuel Cell Fabrication

A similar deposition process was used to coat the gas diffusion electrode (GDE) supports used for fuel cell fabrication with the S-PEI and S-GO multilayers. Two layers of S-PEI and six layers of S-GO were deposited on a GDE. A 12-μm PFSA membrane was placed between a coated GDE and an uncoated GDE. The stack was then laminated to complete a fuel cell membrane-electrode assembly (MEA). A subgasket made of poly­(ethylene naphthalate) sheets was used to protect the edges of the MEA. The MEA active area was 5 cm^2^. The electrode Pt loadings were 0.2 mg/cm^2^ in all cases. The MEA fabrication process was similar to that reported previously.^31^

### Fuel Cell Testing

All MEAs were conditioned for 14 h prior to testing in order to humidify and clean the MEAs. The electrochemically active surface area (ECSA) of Pt was measured by integrating the underpotentially deposited hydrogen adsorption and desorption (HAD) peaks in a cyclic voltammogram at 30 °C in fully humidified H_2_/N_2_. H_2_ permeance tests were performed by measuring the diffusion-limiting current (0.35 V) in a H_2_/N_2_ cell. Different concentrations of H_2_ diluted in N_2_ were flowed into the anode compartment. H_2_ permeance was determined from the slope of the crossover current and H_2_ concentrations. All reported electrochemical measurements are averages of more than three replicates with uncertainties reported as standard deviations. All voltages are reported with respect to the reversible hydrogen electrode.

### Optical Null Ellipsometry

Layer thicknesses were determined using a rotating optical ellipsometer (M-44, J. A. Woollam Co., Inc.) using 44 discrete wavelengths between 400 and 750 nm simultaneously. WVASE32 software (Woollam) was used for data acquisition and reduction.

### UV–Visible Spectroscopy

A Cary model 4000 UV–visible spectrometer was used for the acquisition of absorption spectra reported in this work. Spectral resolution was set to 2 nm for all measurements.

### X-ray Photoelectron Spectroscopy

The University of Michigan Center for Materials Characterization performed XPS measurements with a Kratos Axis Supra+ instrument. Samples were interrogated using a monochromatic Al kα X-ray beam at 1.486 keV with anode settings of 15 kV and 20 mA. The sample spot size for data acquisition was ∼700 μm × 300 μm at photoelectron pass energies of 160 eV (survey scans) and 20 eV (core scans). spectral acquisition step size was 0.1 eV (core scans) and 1 eV (survey scans).

### Scanning Electron Microscopy

SEM images were acquired using A JEOL 7500F (field emission emitter) scanning electron microscope (JEOL Ltd., Tokyo, Japan) with 5.0 kV accelerating voltage. Image processing and analysis were performed using SMILE VIEW Map software (JEOL). Samples were coated with ∼10 nm of Os using a chemical vapor deposition (CVD) coater (Tennant20, Meiwafosis Co., Ltd.) and were mounted on standard Al stubs with carbon suspension cement (SPI Supplies) and epoxy glue (System Three Quick Cure 5).

## Results and Discussion

Controlling the properties of ultrathin films and coatings on different surfaces has posed challenges to the chemistry and materials communities for years. A useful structural motif has been the growth of interfaces with well-resolved control over the distance of specific functionalities from the support surface. We report on the growth of hybrid multilayers here, where the LbL growth of the polymer and the graphene oxide proceeds with monolayer thickness resolution. The PEI we have used is branched, and that structural property bears on the extent to which these polymer layers can be uniformly deposited onto a nonuniform substrate. The lateral integrity and uniformity of the deposited PEI layer is related to the branched structure of the polymer, and the relevant length scale of the lateral uniformity is expected to be at least similar to and likely greater than the dimensions of the GO moieties subsequently deposited on the PEI adlayer.

We have constructed interfaces where we deposit initial adlayer(s) of S-PEI onto a silica support using Zr^4+^ complexation chemistry, and subsequently, have deposited adlayers of S-GO using the same Zr^4+^ complexation chemistry. In all cases we obtain linear adlayer growth for the GO layers and the initially deposited PEI monolayer is of thickness similar to that of the subsequent S-GO adlayers. We show the ellipsometric data for these multilayer structures in Figure 3.

**Figure 3:**
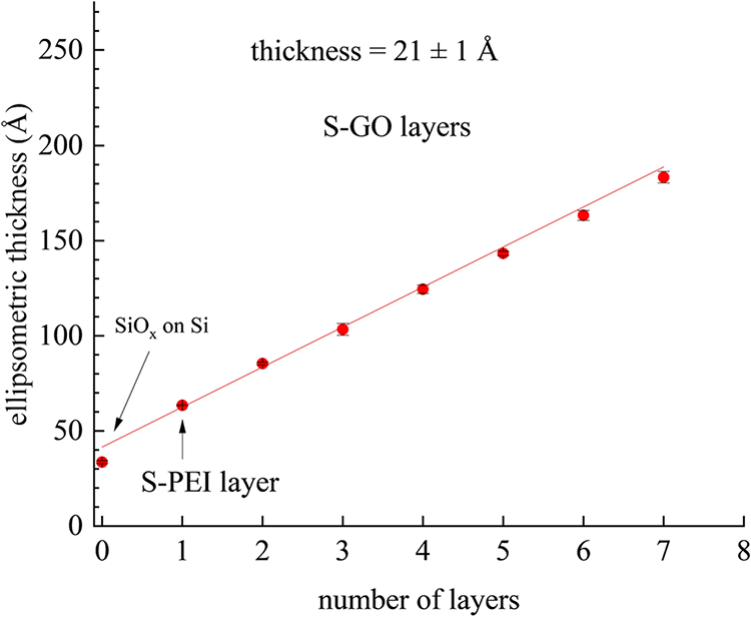
Ellipsometric thickness data for S-PEI monolayer with six S-GO monolayers.

The S-GO layer thickness is consonant with molecular mechanics calculations assuming a 10 Å thickness for a GO layer, with the average thickness being 21 ± 1 Å/layer. While the Zr­(XSO_3_)_2_ complex free energy of formation is not known to the best of our knowledge, our experimental observations indicate that it is lower in strength than that of the better-known Zr­(XPO_3_)_2_.^32^

In addition to ellipsometric data, it is useful to examine these multilayer structures using UV–visible absorbance measurements because these data may provide insight into the linearity of growth. We performed absorbance measurements on multilayers deposited on fused silica (Figure 4).

**Figure 4:**
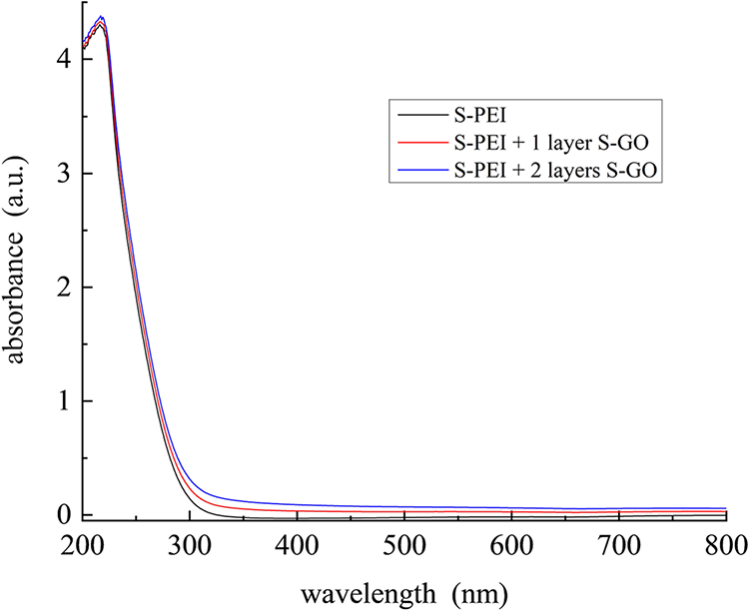
UV–visible absorbance spectra of S-PEI and S-GO adlayers (2) on a silica substrate.

The absorbance data for the S-PEI and S-PEI + S-GO adlayers exhibit a prominent absorption band at *ca*. 240 nm, which we assign to the XSO_3_ functionality. This band is not seen for phosphate-modified-GO adlayers, where no prominent feature is observed for wavelengths longer than 200 nm.^29^ For the S-GO adlayers, the scattering contribution to the data dominates for wavelengths longer than *ca*. 325 nm. The data for the S-PEI + S-GO adlayers are thus of limited utility in gauging uniformity of layer deposition owing to the prominence of the RSO_3_ absorbance band. The absorbance data are consistent in terms of adlayer deposition with the ellipsometric data.

It is useful to consider the stoichiometric relationship between Zr and S in our samples because this information speaks to the nature of the interlayer complexation. We find that for the S-PEI + S-GO multilayer assembly, the S/Zr ratio is 12.5 ± 2.8, suggesting incomplete complexation of the nominally available sulfate groups. This result is consistent with energetically moderately favorable complexation between Zr^4+^ and sulfamates/sulfates or sulfates/sulfates, and with our previous reports on the synthesis and layer growth of modified GO using Zr-sulfamate and Zr-phosphate layer bonding chemistry.^29,30^

It is important to understand the morphology of the hybrid multilayers on length scales beyond molecular dimensions. SEM data (Figure 5) provide an effective means of evaluating thin film morphology. The cross-sectional images of the S-GO adlayers appear to be regular. The top view of the sulfated layer appears to be uniform and devoid of prominent features. This may be due to the ability of the Zr^4+^ ions and the sulfate groups to anneal, resulting in a relatively uniform layer, consistent with the molecular-scale information contained in the ellipsometry and XPS data.

**Figure 5:**
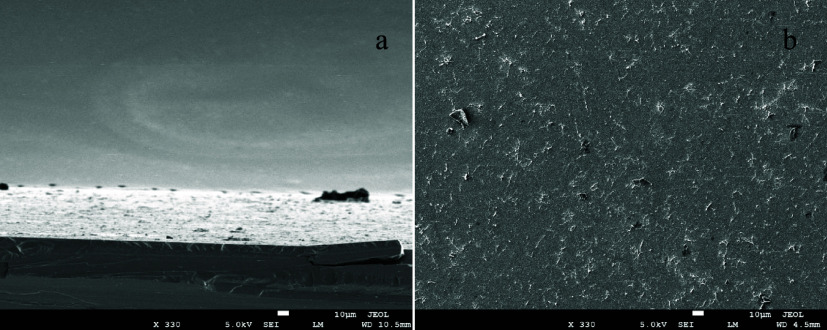
SEM images of the (S-PEI + S-GO) multilayers on a Si substrate. (a, b) correspond to the cross section and surface (top down) image of the (S-PEI + S-GO) layers. These images are of 8 layers total (2 layers of modified PEI and 6 layers of modified GO).

Following characterization of these hybrid multilayers, it is important to consider their practical utility. As a demonstration of their utility, the effects of six layers of S-GO were studied in a PEM fuel cell. As shown in Figure 6a, in the absence of the initial S-PEI adlayer, S-GO adlayers deposited onto the GDE support did not provide any detectable resistance to H_2_ gas permeation for up to 30 layers of deposited S-GO. SEM data suggest that the deposition of S-GO was nonuniform and not continuous. In other words, the characteristic dimensions of the S-GO moieties were not sufficient to span the openings and voids that characterize the GDE support. Likewise, the two adlayers of S-PEI by themselves did not provide any resistance to H_2_ permeation. However, when both S-PEI and S-GO were deposited, a ∼15% reduction of H_2_ gas crossover was observed. This finding indicates that the S-PEI adlayers possess the lateral integrity to span nonuniformities in the GDE support, while the S-GO adlayers serve to limit H_2_ permeation. Previous work showed that a 60 nm GO layer reduced H_2_ gas crossover of a 12-μm PFSA membrane by ∼50%.^31^ Its H_2_ permeability was estimated to be 1.2 × 10^–15^ cm·mol/(cm^2^·s·kPa) or a factor of 240 smaller than PFSA ionomer. Using this permeability, a 12 nm S-GO layer is expected to reduce H_2_ gas crossover by 17%, in good agreement with the result we report here. Figure 6b compares the fuel cell polarization curves with different layers on the anode GDEs. Comparing to the uncoated baseline MEA, the MEA with S-PEI + S-GO adlayers showed about 5 mV and 25 mV lower voltage at low and high current density regions. There was no increase in proton resistance observed to within the uncertainty of our measurements. The voltage gap can potentially be due to trace levels of contaminants or increased mass transport resistance. It should also be noted that this level of voltage gap is not uncommon for small scale R&D testing at this stage. This potential issue will be investigated in more detail in the future.

**Figure 6:**
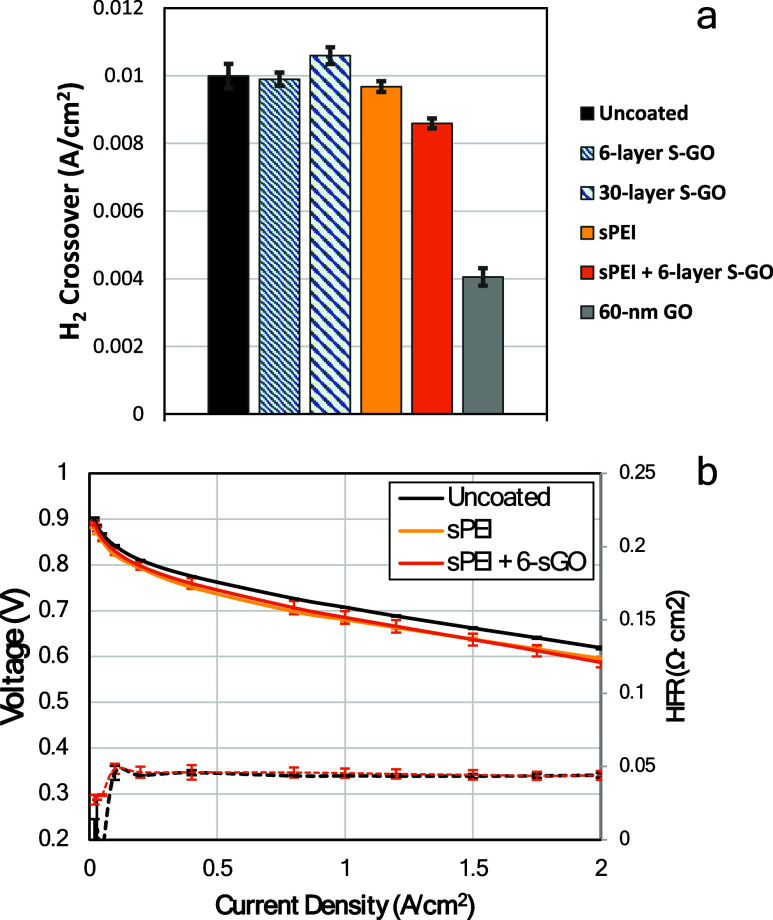
(a) H_2_ crossover current measured at 80 °C and 95% relative humidity, 245 kPa_abs_. (b) H_2_/air fuel cell performance curve of MEAs with and without layers at the anode-membrane interface. 94 °C, 90/90% RH, 250/250 kPa_abs_, high stoichiometries. Results from three individual cells with 5 cm^2^ active area are reported.

## Conclusions

We have designed and characterized six layers of sulfated graphene oxide deposited on sulfamated PEI using Zr-sulfate interlayer linking chemistry. The deposition is characterized by relatively fast adsorption/bonding kinetics, as seen by the time scales required to form adlayers, and each layer can be formed by exposure to the solutions containing functionalized PEI and GO species for each layer. Optical ellipsometry and absorbance data show layer-by-layer growth with layer thicknesses consistent with expectations. SEM images reveal the formation of relatively homogeneous layers. Demonstration of the utility of these hybrid LbL materials shows that the use of a polymer supporting layer can successfully mediate structural nonuniformities in support structures and allow molecular-scale chemical processes intrinsic to GO to be realized with real-world supporting materials. We anticipate that these hybrid multilayers will also find use in the design of chemically selective surfaces for other application areas.

## Supplementary Material



## Notes

FTIR spectra of PEI and S-PEI, XPS data for S-PEI + S-GO and SEM data for S-GO modified supports without S-PEI modification (PDF)
