# Supplementary material for: Design and Characterization of Hybrid Multilayer Structures: Layer-by-Layer Growth of Polymer and Graphene Oxide Assemblies and Their Utility in Fuel Cell Applications
Source: ACS Appl Energy Mater. 2026 Jan 13;9(2):1228–33. doi: 10.1021/acsaem.5c03672 (PMC12848851; doi:10.1021/acsaem.5c03672)
Supplement: Supplementary file 1 [file ae5c03672_si_001.pdf]

Supporting Information

Design and Characterization of Hybrid Multilayer Structures: Layer-by-Layer Growth of Polymer and Graphene Oxide Assemblies and their Utility in Fuel Cell Applications

Neelanjana Mukherjee,[1] Nancy S. Muyanja[2] , Yunzhu Zhang[3], Phuong Quynh Ngo[3], Anusorn Kongkanand[3], and G. J. Blanchard[1,*]

1 Michigan State University, Department of Chemistry, 578 S. Shaw Lane, East Lansing, MI 48824 USA
2 Michigan Center for Materials Characterization, University of Michigan, College of Engineering, Ann Arbor, MI 48109 USA
3 Fuel Cell Businesses, General Motors, Pontiac, MI 48340 USA

Contents

(1) FTIR spectra of Polyethylene imine (PEI) and Sulfamated polyethylene imine (S-PEI)
(2) Listing of relevant bands for S-PEI in tabular format
(3) XPS survey scan of S-PEI + S-GO adlayers
(4) XPS table of sample composition.
(5) SEM images of six and thirty layers of Zr-S-GO on porous supports that have not been modified with S-PEI, and corresponding energy-dispersive X-ray elemental analyses.

[*] Author to whom correspondence should be addressed: email: blanchard@chemistry.msu.edu, Tel: +1 517 353 1105

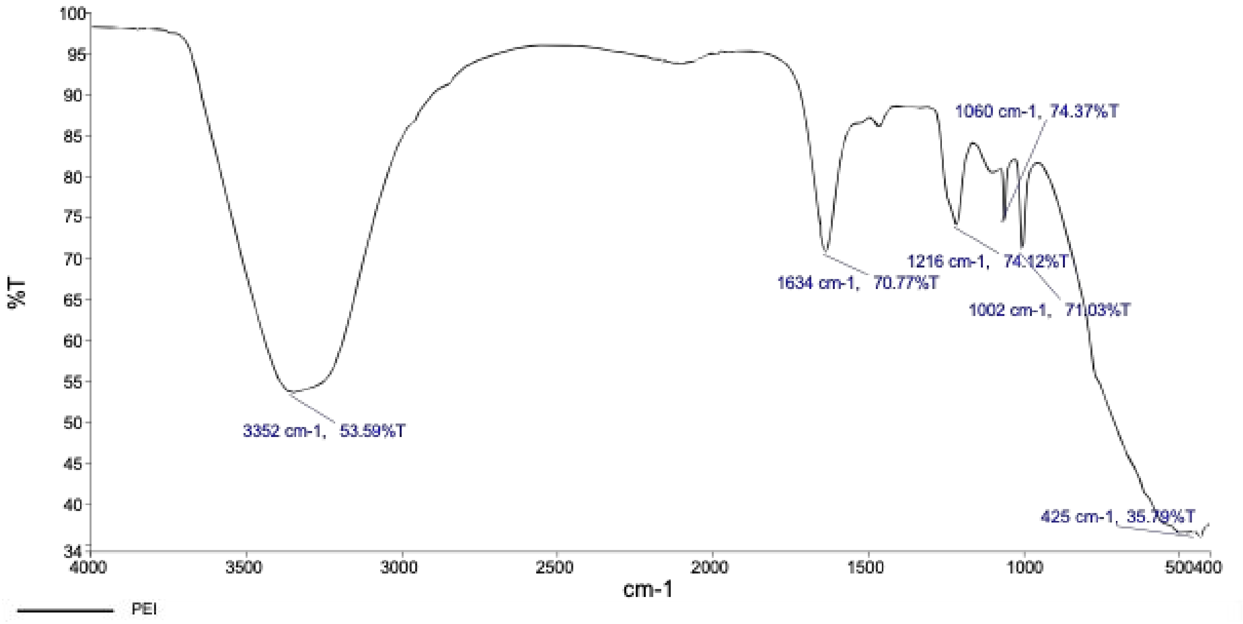

Figure S1a: FTIR spectra of PEI.

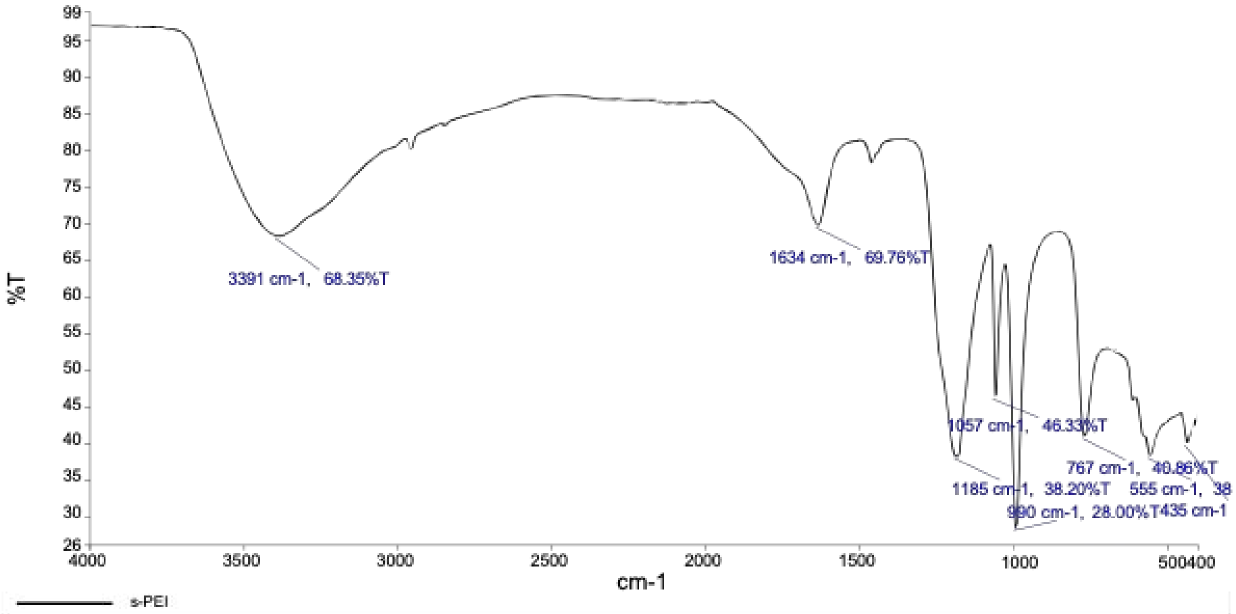

Figure S1b: FTIR spectra of sulfamated PEI (S-PEI).

Table S1: Band assignments and observed frequencies for S-PEI.

literature frequency (cm^{-1})	observed frequency (cm^{-1})	Band Assignment
990	991	S=O sym stretch
1056, 1190	1057, 1185	S=O asym stretch
590	555	S-O stretch
1641	1634	C=C stretch

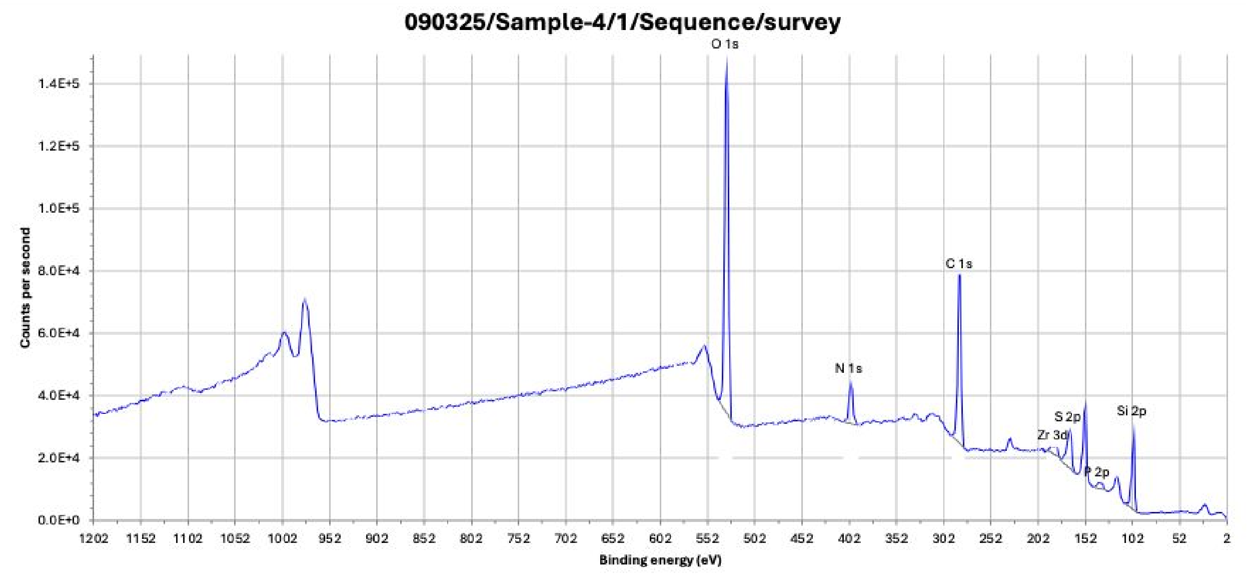

Figure S2: XPS spectra of the P-PEI and S-PEI samples

Table S2. XPS atomic concentrations (%) for Zr, P, S, C and O. Uncertainties are ±1σ. XPS band assignments made using vendor software.

Sample	Zr 3d	P 2p	S 2p	C 1s	O 1s
s-PEI + s-GO	0.4±0.1	0.5±0.1	5.7±0.3	41±0.5	30.2±0.3

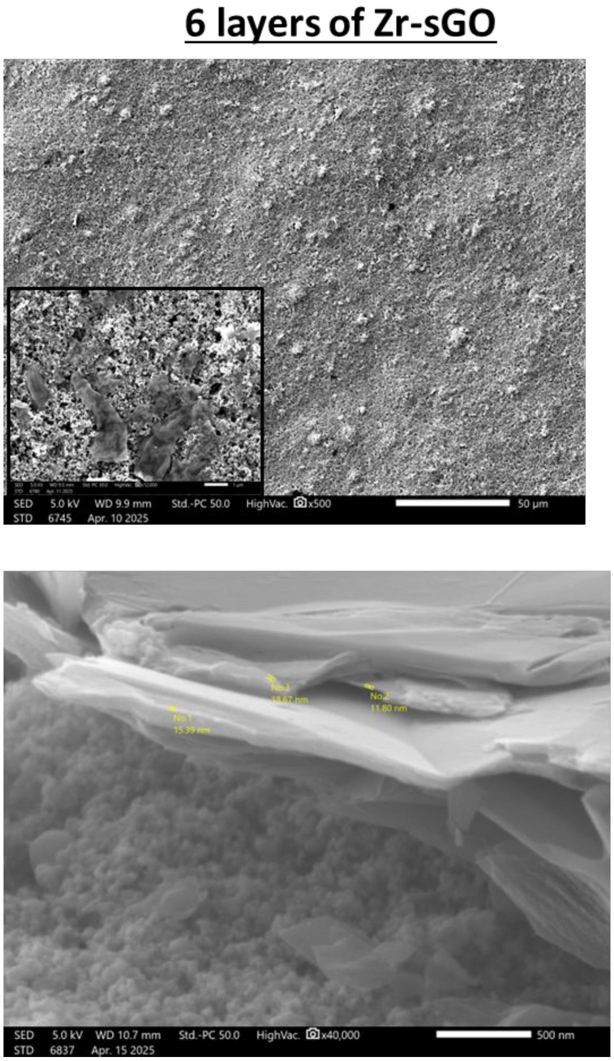

Figure S3. SEM images of six and thirty layers of Zr-S-GO on porous supports that have not been modified with S-PEI. Top images are top views, and bottom images are side-on views, showing thicknesses of S-GO-modified areas. For these non-modified supports the GO coverage appears to be non-uniform.

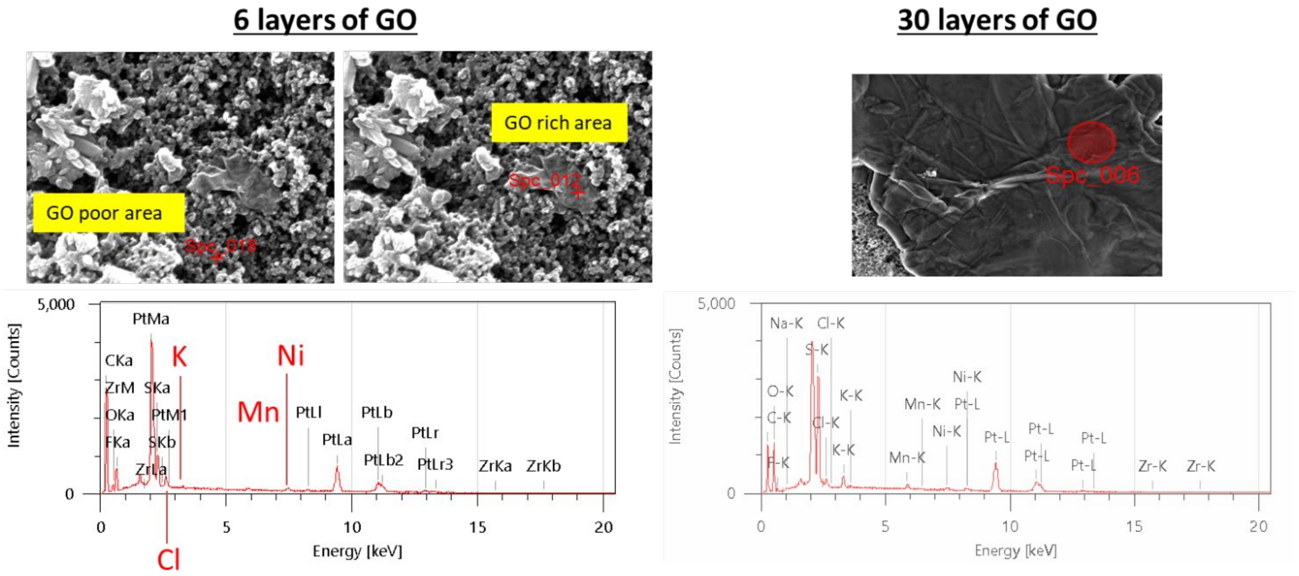

Element	Line	Mass%	Atom%
C	K	63.96±0.36	83.17±0.47
O	K	6.40±0.25	6.25±0.25
F	K	10.16±0.24	8.35±0.20
S	K	1.63±0.03	0.79±0.02
Zr	L	nd	nd
Pt	M	17.85±0.15	1.43±0.01
Total		100.00	100.00
Spc_018	Fitting ratio 0.0490		

Element	Line	Mass%	Atom%
C	K	43.82±0.36	62.15±0.52
O	K	28.75±0.44	30.61±0.46
F	K	1.92±0.13	1.72±0.11
Na	K	0.20±0.02	0.15±0.02
S	K	5.92±0.06	3.15±0.03
Cl	K	0.37±0.02	0.18±0.01
K	K	0.60±0.02	0.26±0.01
Mn	K	0.43±0.02	0.13±0.01
Ni	K	0.38±0.03	0.11±0.01
Zr	K	0.11±0.11	0.02±0.02
Pt	L	17.51±0.23	1.53±0.02
Total		100.00	100.00
Spc_006	Fitting ratio 0.0176		

Figure S4. Top: SEM images of selected regions of the samples shown in Fig. S3, underscoring heterogeneity in the distribution of S-GO on the support that has not been modified with S-PEI prior to S-GO deposition. Middle: Energy-dispersive X-ray elemental analysis of the spots indicated on the images. Bottom: Tables of elemental compositions of the measured regions. Penetration depth of the e-beam is estimated to be on the order of 1 μm.

References

Y. Zhang, X. Li, Z. Cao, Z. Fang, T. R. Hull and A. A. Stec. Synthesis of Zinc Phosphonated Poly(ethylene imine) and Its Fire-Retardant Effect in Low-Density Polyethylene. *Ind. Eng. Chem. Res*. 2015, **54**, 3247−3256. DOI: 10.1021/ie504200y

D. M.G. Saad, E. M. Cukrowska and H. Tutu. Sulfonated cross-linked polyethylenimine for selective removal of mercury from aqueous solutions, *Toxicological and Environmental Chemistry*, 2012, **94:10**, 1916-1929. DOI: 10.1080/02772248.2012.736997
